# Polysaccharide-Based Injection Matrix for Serial Crystallography

**DOI:** 10.3390/ijms21093332

**Published:** 2020-05-08

**Authors:** Ki Hyun Nam

**Affiliations:** Department of Life Science, Pohang University of Science and Technology, Pohang 37673, Korea; structures@postech.ac.kr

**Keywords:** serial crystallography, sample delivery, viscous medium, wheat starch, alginate, SMX, SFX, background scattering

## Abstract

Serial crystallography (SX) provides an opportunity to observe the molecular dynamics of macromolecular structures at room temperature via pump-probe studies. The delivery of crystals embedded in a viscous medium via an injector or syringe is widely performed in synchrotrons or X-ray free-electron laser facilities with low repetition rates. Various viscous media have been developed; however, there are cases in which the delivery material undesirably interacts chemically or biologically with specific protein samples, or changes the stability of the injection stream, depending on the crystallization solution. Therefore, continued discovery and characterization of new delivery media is necessary for expanding future SX applications. Here, the preparation and characterization of new polysaccharide (wheat starch (WS) and alginate)-based sample delivery media are introduced for SX. Crystals embedded in a WS or alginate injection medium showed a stable injection stream at a flow rate of < 200 nL/min and low-level X-ray background scattering similar to other hydrogels. Using these media, serial millisecond crystallography (SMX) was performed, and the room temperature crystal structures of glucose isomerase and lysozyme were determined at 1.9–2.0 Å resolutions. WS and alginate will allow an expanded application of sample delivery media in SX experiments.

## 1. Introduction

Traditional X-ray crystallography plays an important role in structural biology, as it allows detailed understanding of chemical and biological functions and, ultimately, the mechanisms underlying them [1,2]. It also provides insight into drug design or rational engineering of industrially relevant enzymes [3,4]. However, it has experimental limitations, including radiation damage, cryogenic temperatures, and static structures [5,6], which can be addressed by the recently emerging serial crystallography (SX) technique [7]. X-ray free electron lasers (XFELs) with ultrafast pulse-duration and synchrotron X-rays reduce radiation damage during data collection [8,9,10,11]. Among them, the SX experiment at synchrotron enables low-dose data collection, since the crystal samples’ X-ray exposure time is shorter than in the conventional crystallographic approach [8]. These techniques can be beneficially applied to observe radiation-sensitive metalloproteins or structural flexibility at room temperature, and time-resolved studies [7,8,12,13,14]. In addition, the intense X-ray facilitates the determination of the crystal structure from crystals of a few microns or smaller. Thus, SX has scientific advantages over traditional X-ray crystallography; however, it requires a large number of crystal samples and a sample delivery method that continuously delivers crystals to the X-ray interaction point [15]. Various sample delivery systems, such as injectors [16,17,18], syringes [19,20,21], fixed target scans [22,23,24,25,26], microfluidic devices [27,28], capillaries [29,30], sample extractors [31], and polyimide tube-based containers (quasi 1D fixed target) [32], have been applied to serial femtosecond crystallography (SFX) or serial millisecond crystallography (SMX) experiments. Among them, the delivery of crystals embedded in a viscous medium produced stable injection streams, even at very low flow rates, and was widely applicable in SX in synchrotrons or XFEL facilities with low repetition rates [33].

Sample delivery media can be classified into either amphiphilic, hydrophobic, or hydrophilic materials [33]. Amphiphilic sample delivery medium, such as lipidic cubic phases (LCPs) [17], and hydrophobic sample delivery media, such as greases [19,34,35,36] and shortening [37], produce a stable injection stream with high viscosity while exhibiting certain background scattering rings from the delivery material. Hydrophilic delivery materials can be further classified into sugar-based media, such as agarose [38], hyaluronic acid (HA) [34], hydroxyethyl cellulose (HEC) [35], and sodium carboxymethyl cellulose (NaCMC) [39], and polymer-based media, such as Pluronic F-127 [39], poly(ethylene oxide) [14], and polyacrylamide (PAM) [40]. Although typical sugar-based delivery media reveal very low levels of X-ray background scattering [34,35,38,39] and have the advantage of improving the signal-to-noise ratio (SNR) when compared with other sample delivery media [34,35,38,39], they can be recognized as substrates by certain protein crystal samples, resulting in biological and chemical reactions that can dissolve or damage crystal samples [33]. For example, agarose or cellulose-derived delivery materials can react with agarase or cellulase crystal samples. Therefore, continuous development of delivery media is necessary for extensive applications in future SX studies.

Starch is the main source of energy for humans and is stored as a carbohydrate reserve in plants [41]. It is almost entirely composed of linear amylose and its branched counterpart, amylopectin [42]. These polysaccharides consist of α-(1,4)-linked D-glucose residue chains interconnected via α-(1,6)-glucosidic linkages [41]. Alginates are abundant in nature and primarily found in marine brown algae or capsular polysaccharides in soil bacteria [43]. They belong to a family of unbranched binary copolymers and consist of (1,4)-linked β-d-mannuronic acid and α-l-guluronic acid chains in widely varying compositions and sequences [43]. Alginates have an almost temperature-independent sol/gel transition in the presence of multivalent cations (e.g., Ca^2+^), which is applicable in the immobilization of living cells [43]. Since these materials have oligosaccharides with different chemical structures than those previously reported, they can provide an expanded opportunity for use as a delivery medium for sugar-binding protein crystals in SX experiments.

Here, the preparation and characterization of wheat starch (WS) and alginate as sample delivery media are introduced for SX experiment. Both delivery media produced stable injection streams at flow rates <200 nL/min and generated very low-level X-ray background scattering. SMX experiments using WS and alginate as delivery media were performed, and the room temperature structures of glucose isomerase and lysozyme were determined at 1.9–2.0 Å resolutions.

## 2. Results

### 2.1. Characterization of the Starch Injection Matrix

Typically, polysaccharides can become gelatinized when the temperature is lowered after swelling at high temperature [44]. Additionally, they can remain viscous via hydrogen bonding and van der Waals interactions between chains [45]. In previous reports, the sugar-based delivery medium, agarose, was used in gel form at lower temperatures following melting at high temperatures [38], whereas highly viscous HA, HEC, and NaCMC were prepared by increasing their concentrations, then used as delivery media [34,35,39]. Based on this background, it was expected that WS and alginate could be used as delivery materials in gel or high-viscosity states.

**Figure 1:**
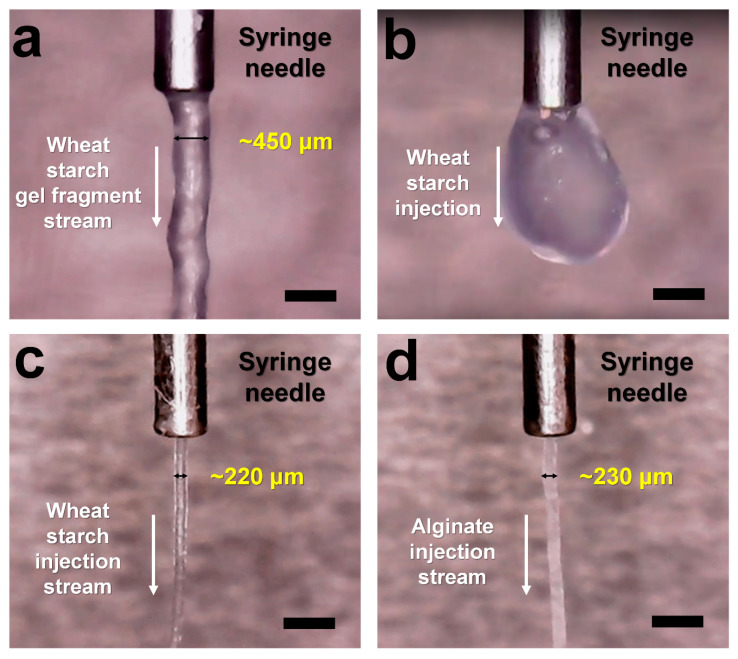
Snapshot of the injection stream of (**a**) 5% (*w*/*v*) WS gel fragment, (**b**) 5% (*w*/*v*) WS, (**c**) 14% (*w*/*v*) WS, and (**d**) 14% (*w*/*v*) alginate. WS and alginate delivery media were extruded from a syringe needle with an inner diameter of 168 μm at room temperature. The scale bar indicates 718 μm.

WS and alginate gels were first prepared by raising and lowering the temperature, then used as delivery materials. WS and alginate (5% *w*/*v*) were melted above 100 °C and left at room temperature to form gels. However, as temperature was lowered, water from the gel evaporated and adhered to the upper tube cap, indicating that the gel had a concentration higher than 5%. Meanwhile, WS and alginate gel structures are difficult to break; hence, mixing them directly to embed the crystal sample into the gel may cause physical damage to the sample, thereby increasing the mosaicity of the crystal and reducing the diffraction intensity. In order to solve this problem, as in the preparation of the PAM injection matrix [40], WS and alginate gels were fragmented, and the crystal samples were embedded onto the gel fragment. After melting the WS and alginate at >100 °C, the WS and alginate solutions were transferred into 100 μL syringes using a pipette and left at room temperature for 10 min. After gelatinization, the WS and alginate gels were mixed and fragmented by pushing the plungers back and forth in a dual syringe setup. Next, these gel fragments were gently mixed with lysozyme crystal samples using the same setup. Crystal samples embedded in WS or alginate gel fragments were able to produce a continuous injection stream through the syringe needle at a flow rate of 200 nL/min (Figure 1a). The behavior of this injection stream is expected to be used to deliver crystal samples; however, as mentioned above, the evaporation of water from the gel during the process of raising and lowering the temperature can change the final gel concentration. Thus, this approach was not considered suitable in terms of reproducibility of the delivery medium preparation. Meanwhile, the previous study used low-melting agarose [38], which may have little effect on the evaporation of water. As a result, the melting temperature of sugar is considered to be important when gelling sugar and using it as a delivery medium.

**Figure 2:**
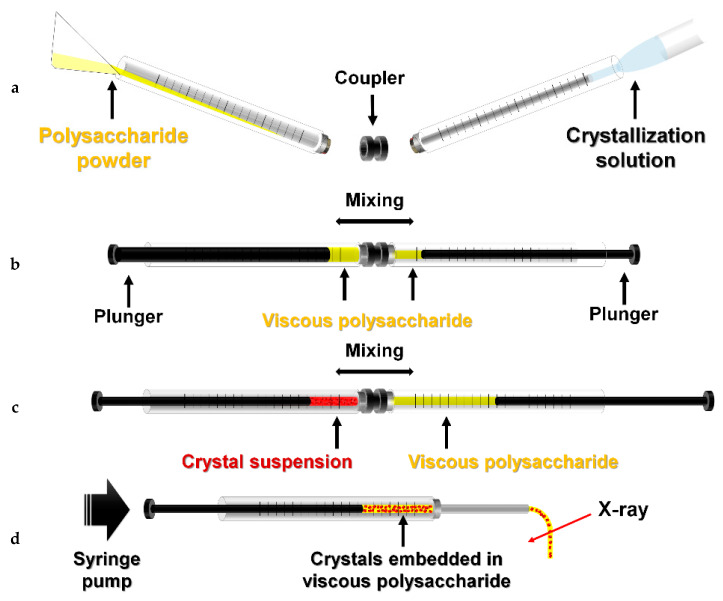
Preparation of WS and alginate delivery media for SX. (**a**) Wheat starch (WS) or alginate powder was added to a 250 μL syringe and crystallization solution to a 100 μL syringe. (**b**) WS or alginate was mixed with crystallization solution in a dual syringe setup. (**c**) Viscous WS or alginate was mixed with the crystal suspension. (**d**) After connecting the syringe needle, crystals embedded in the viscous medium were delivered to an X-ray interaction point using a syringe pump.

Next, highly viscous WS and alginate were prepared by increasing the concentrations, then used as delivery media. During sample preparation, it was difficult to transfer highly viscous WS and alginate prepared in microcentrifuge tubes into syringes using pipettes, and the use of spatulas would lead to large sample losses. Therefore, as previously reported for the preparation of the poly(ethylene oxide) (PEO) delivery medium [14], WS or alginate powder was added directly into a 250 μL syringe with an internal diameter of 2.3 mm, and the crystallization solution to dissolve the powder was placed in a 100 μL syringe (Figure 2a). Both syringes were connected to the coupler then mixed back and forth > 50 times using a plunger (Figure 2b). The viscous delivery medium was transferred to the 100 μL syringe, and the 250 μL syringe was removed. The 100 μL syringe containing the crystal suspension was connected to the syringe containing the delivery medium and gently mixed back and forth 30 times using the plunger (Figure 2c). The crystal samples embedded in the delivery medium were transferred to one syringe, and the partner syringe and coupler were removed. After connecting the needle to the syringe containing the crystals, the delivery medium containing crystal samples was delivered to an X-ray interaction point using a syringe pump (Figure 2d).

WS or alginate (5–20% *w*/*v*) was prepared, and the injection stability was initially screened using a syringe with a 168 μm inner diameter (ID) needle at a flow rate of 200 nL/min. At WS and alginate concentrations less than 8%, the injection stream was unstable or often presented drop-like formations at the tip of the needle (Figure 2b). WS and alginate showed stable injection streams at concentrations above 12%. In this SMX experiment, a 20% (*w*/*v*) WS or alginate delivery medium was mixed with the crystal suspension in a 3.5:1.5 ratio in a dual syringe setup, and the final 14% (*w*/*v*) WS or alginate delivery medium was used in the SMX experiment. The diameters of the injection stream flows of WS and alginate were ~220 and ~230 μm, respectively, despite passing through a needle with a 168 μm inner diameter (Figure 2c,d).

### 2.2. Measurement of Background Scattering

**Figure 3:**
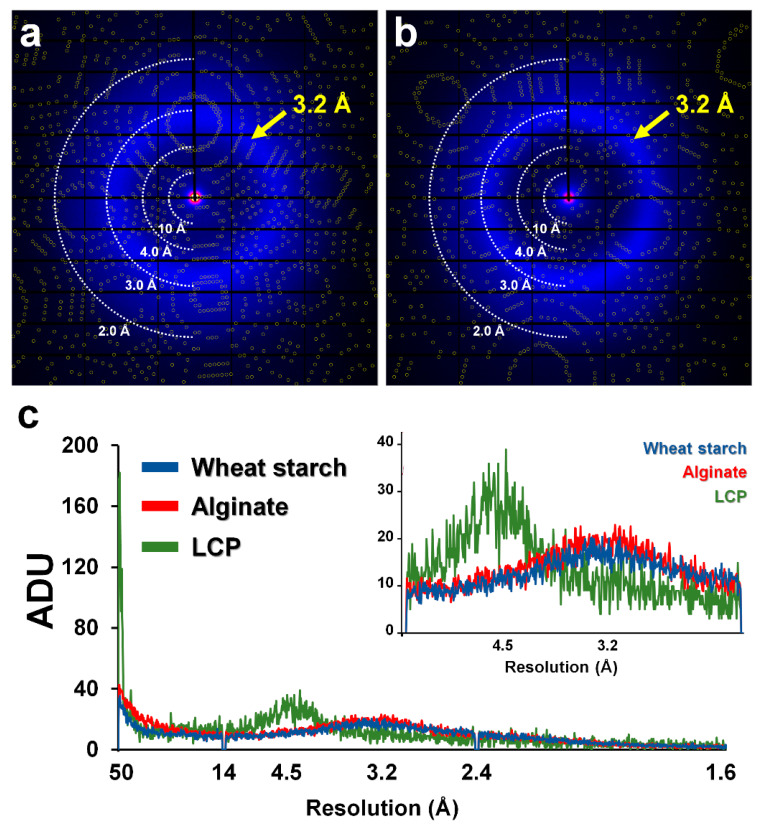
Measurement of X-ray background scattering of WS and alginate. Diffraction pattern of glucose isomerase embedded in 10% (*w*/*v*) (**a**) WS and (**b**) alginate delivery media. The circles show predicted positions of Bragg peaks by CrystFEL. (**c**) 2D profile of the average scattering of WS (blue), alginate (red), and LCP (green). (Inset) Magnified view of background scattering in the range 14–2.4 Å. Yellow arrows indicate 3.2 Å resolution area.

X-ray exposed crystal embedded sample delivery could produce a diffraction pattern of the crystal sample, as well as background scattering of the delivery material [33]. X-ray background scattering of the delivery medium could affect the SNR and diffraction data quality; hence, it is an important criterion during the selection of delivery media for SX experiments [33,38]. Since the viscous state of WS and alginate maintains unregulated hydrogen bonds between polysaccharides, it is expected to show very weak or no scattering that does not produce significant scattering at certain angles. To prove this, the X-ray background scattering of the WS and alginate delivery media was measured. The 14% (*w*/*v*) WS and alginate injection streams were delivered to X-rays via a syringe needle with a 168 μm ID. The diameters of WS and alginate injection streams were approximately 220 μm and 230 μm, respectively. Average intensities were analyzed from the beam stopper to 1.6 Å resolution. The highest intensity, approximately 40 ADU (analog-to-digital unit), was observed at 100 Å around the beam stopper for both the WS and (Figure 3a) and alginate (Figure 3b) delivery media. Moreover, both media showed diffused scattering of < 20 ADU in the 3.2–3.4 Å region, which is considered as solvent scattering from the crystals and the delivery media. Next, the background scattering of WS and alginate was compared with LCP delivery medium, which is widely used in SFX research. The diameter of LCP used for background scattering measurements was approximately 190 μm, which was delivered from a syringe with a 168 μm ID. LCP showed high background scattering of > 170 ADU at 100 Å around the beam stopper and also produced >30 ADU levels of background scattering at 4–4.5 Å, which is considered as scattering from lipid packing (Figure 3c). LCP showed higher intensity for background scattering around 4.5 Å resolution than WS or alginate, while it was lower than WS or alginate around 3.2 Å resolution. Not surprisingly, the LCP used in the experiment was composed of 60% (*w*/*v*) monoolein, whereas WS and alginate had a concentration of 14% (*w*/*v*). In other words, the proportion of solvent in the LCP injection stream was 40%, whereas the proportion of solvent in the WS and alginate injection streams was 86%; thus, the higher water scattering intensity at 3.2 Å for WS and alginate compared to LCP was expected. Nevertheless, overall background scattering was lower for the WS and alginate injection streams than for LCP.

### 2.3. Application of WS and Alginate Injection Media for SMX

To demonstrate that WS and alginate can be applied to SX experiments as sample delivery media, SMX experiments were performed at a synchrotron (Table 1). Glucose isomerase (GI) and lysozyme crystals were embedded in the WS or alginate, and they were extruded from syringe needle with a 168 μm ID to X-ray beam path at a flow rate of 200 nL/min at room temperature. 

For GI embedded in WS, a total of 81,798 images were collected, and hit images, including diffraction patterns, totaled 8361 images with a hit rate of 10.22%. The data processing resulted in a GI diffraction pattern from a total of 7732 images with an indexing rate of 92.47% (Figure 3A). In the indexed image, a total of 8281 diffraction patterns, including multi-crystal hit patterns, were obtained. Data were processed up to 2.0 Å, with completeness, SNR, R_split_, and CC* of 100, 4.01, 17.22, and 0.9937, respectively. R_work_ and R_free_ of the final model structure were 17.05% and 21.30%, respectively. For lysozyme embedded in WS, a total of 34,484 images were collected, and hit images, including diffraction patterns, totaled 4231 images at a hit rate of 12.27%. The data processing resulted in a lysozyme diffraction pattern from a total of 3789 images with an indexing rate of 89.55%. In the indexed image, a total of 4383 diffraction patterns, including multi-crystal hit patterns, were obtained. Indexed diffraction data were processed up to 2.0 Å, with completeness, SNR, R_split_, and CC* of 100, 4.04, 17.06, and 0.9907, respectively. R_work_ and R_free_ of the final model structure were 18.41% and 23.33%, respectively. 

For GI embedded in alginate, a total of 25,000 images were collected, and hit images, including diffraction patterns, totaled 10,268 images with a hit rate of 41.07%. The data processing resulted in a GI diffraction pattern from a total of 9946 images with an indexing rate of 96.86% (Figure 3B). In the indexed image, a total of 11,289 diffraction patterns, including multi-crystal hit patterns, were obtained. Data were processed up to 2.0 Å, with completeness, SNR, R_split_, and CC* of 100, 4.19, 18.99, and 0.9953, respectively. R_work_ and R_free_ of the final model structure were 19.23% and 22.58%, respectively. For lysozyme embedded in alginate, a total of 4563 images were collected, and hit images, including diffraction patterns, totaled 3968 images at a hit rate of 86.96%. The data processing resulted in a lysozyme diffraction pattern from a total of 1506 images with an indexing rate of 37.95%. In the indexed image, a total of 2066 diffraction patterns, including multi-crystal hit patterns, were obtained. Indexed diffraction data were processed up to 1.9 Å, with completeness, SNR, R_split_, and CC* of 100, 3.91, 17.70, and 0.9916, respectively. R_work_ and R_free_ of the final model structure were 18.96% and 23.82%, respectively. 

**Figure 4:**
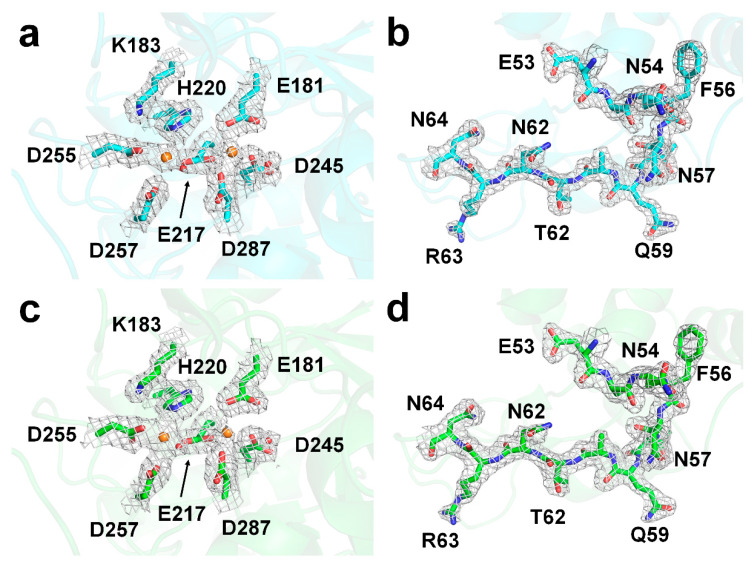
2Fo-Fc electron density map (grey mesh, 1.2 σ) of the active sites of (**a**) glucose isomerase and (**b**) lysozyme delivered in WS (cyan), and (**c**) glucose isomerase and (**d**) lysozyme delivered in alginate (green).

The electron density maps for the entire protein structure were clearly visible (Figure 4). In lysozyme delivered in WS and alginate, no negative fo-fc electron density maps were assumed to have significant radiation damage in four disulfide bonds (C24-C145, C48-C133, C82-C98, and C94-C112). The active site of GI contains two metal-binding sites, which are important for substrate recognition and enzyme activity [46,47]. For GI delivered in WS and alginate, there was no significant negative fo-fc electron density map, indicating no significant radiation damage. 

## 3. Discussion

Here, the preparation and characterization of WS and alginate delivery media were reported for SX experiments. Although alginate performed well as a delivery medium in this experiment, it could form a sol/gel and may be unsuitable as a delivery material when Ca^2+^ is present in the crystallization solution. The chemical structures of sugar-based delivery media reported to date are shown in Table 2. When selecting a sugar-based delivery material, it may be possible to first consider whether chemical interactions between the target protein and the selected delivery material exist based on the chemical structure of the delivery material. To confirm crystal damage caused by the interaction between selected polysaccharide and the crystal sample, a mixture of the crystal sample and the delivery medium was incubated at room temperature for several hours, followed by a crystal morphology evaluation under a microscope or by crystal diffraction quality evaluation by exposing it to X-rays. If the reported polysaccharide-based delivery medium is unsuitable due to specific or nonspecific interaction with the target protein, it is possible to prepare a new viscous delivery material using a polysaccharide substance composed of non-interacting sugars. Although the degree of viscosity varies depending on individual polysaccharide properties, general linear polysaccharides, such as WS, alginate, HA, HEC, and NaCMC, form viscous states by increasing the hydrogen bonds between sugar chains with increasing concentration [45]. These new viscous sugars also present very low levels of background scattering without scattering at certain angles because they exist as random hydrogen bonds between polysaccharide chains. Meanwhile, the crystallization solution of the crystal samples in the practical experiment will be diverse. In actual experiments, the interaction between a specific composition of the crystallization solution and the selected polysaccharide-based delivery material can produce an unstable injection stream. In this case, other polysaccharide-based delivery materials, which do not react with a crystal sample and are not affected by the crystallization solution’s viscosity, may be alternatively used. Therefore, this study extends the pool of viscous crystal delivery media useful in SX experiments.

## 4. Materials and Methods 

### 4.1. Protein Crystal Preparation

GI from *Streptomyces rubiginosus* was purchased from Hampton Research (HR7-098, Aliso Viejo, CA, USA), and was supplied in crystalline form and directly used for the SX experiment without post-crystallization, as previously reported [24]. The size and density of GI were < 60 × 60 × 40 μm^3^ and 3 × 10^7^ crystals/mL, respectively. Lysozyme from chicken egg white was purchased from Sigma-Aldrich (L6876, St. Louis, MO, USA) and crystallized as previously reported [40]. The size and density of lysozyme were < 60 × 60 × 40 μm^3^ and 3 × 10^7^ crystals/mL, respectively.

### 4.2. Preparation of Starch and Aginate Injection Matrix 

Unmodified wheat starch (WS, S5127) and alginate (A2033) were purchased from Sigma-Aldrich (St. Louis, MO, USA). WS (14 mg) or alginate powder (14 mg) was added to a 250 μL syringe (Hamilton, 81065, Reno, NV, USA) and connected via coupler with another 100 μL syringe (Hamilton, 81065) containing crystallization solution (70 μL). The syringe plungers were moved gently back and forth more than 50 times. After removing the 250 μL syringe, the 100 μL syringe containing the protein crystal suspension (30 μL) was connected via a coupler to a 100 μL syringe containing the delivery medium. The syringe plungers were moved gently back and forth more than 20 times, and the crystals were embedded in delivery medium. After moving the mixture to one syringe, the coupler and empty syringe were removed, and the syringe needle was connected. The syringe containing the crystals embedded in WS or alginate was installed into a Fusion Touch 100 syringe pump (CHEMYX, Stafford, TX, USA), and the plunger was pushed through the motor drive to deliver the sample [21].

### 4.3. Data Collection

SMX experiments using a WS injection matrix were performed at the 11C beamline, PLS-II, Pohang, Korea [48]. The X-ray beam size at the sample position was approximately 4 (vertical) × 8 (horizontal) μm^2^ (FWHM). The photon flux was 1.3 × 10^12^ photons/s, and the wavelength was 0.9795. Crystals embedded in WS or alginate were delivered via a syringe pump in horizontal direction to avoid beamline interference [21]. The inner diameter of the syringe needle was 168 μm, and the sample was delivered at a flow rate of 200–300 nL/min. Crystals were X-ray exposed for 100 ms. Diffraction images were corrected on a Pilatus 6M with 10 Hz readout at room temperature.

### 4.4. Data Processing and Structure Determination

Images containing the diffraction pattern were filtered using the Cheetah program [49]. The diffraction pattern of the hit image was processed using CrystFEL [50]. The phases of GI and lysozyme were obtained by molecular replacement using phase-MR in Phenix [51], using the crystal structures of GI (PDB code 5ZYD) [47] and lysozyme (PDB code 6IG6) [40] as the search models. The model was built using COOT [52], individual proteins were refined using Phenix.refinement in PHENIX [53], geometry was validated using MolProbity [54], and all structural figures were produced with PyMOL (DeLano Scientific LLC, San Carlos, CA, USA). The structure factor and coordinate files have been deposited in the Protein Data Bank (Available online: www.rcsb.org/) under the PDB code 7BVL (glucose isomerase delivered in WS), 7BVM (lysozyme delivered in WS), 7BVN (glucose isomerase delivered in alginate), and 7BVO (lysozyme delivered in alginate). Diffraction images have been deposited to CXIDB under ID 124 (glucose isomerase delivered in WS), 125 (lysozyme delivered in WS), 126 (glucose isomerase delivered in alginate), and 127 (lysozyme delivered in alginate).

### 4.5. Background Scattering Analysis

WS, PAM, and LCP (60% (*w*/*v*) monoolein) media were delivered to the X-ray interaction point through a syringe needle with a 168 μm ID. The photon flux of X-rays was 1.3 × 10^12^ photons/s, and exposure was for 100 ms. Background scattering of the delivery media was analyzed using ADXV (Available online: https://www.scripps.edu/tainer/arvai/adxv.html).

## Figures and Tables

**Table 1:** X-ray data collection and refinement statistics.

Data Collection	Glucose Isomerase Embedded in WS	Lysozyme Embedded in WS	Glucose Isomerase Embedded in Alginate	Lysozyme Embedded in Alginate
Energy (eV)	12,659			
Expose time	100 ms			
Space group	I222	P4_3_2_1_2	I222	P4_3_2_1_2
Cell dimension (Å)				
a	94.49	79.36	94.09	79.45
b	100.19	79.36	99.88	79.45
c	103.40	38.63	103.00	38.47
Collected images	81,798	34,484	25,000	4563
Hits images	8361	4231	10,268	3968
Indexed images	7732	3789	9946	1506
Indexed pattern	8281	4383	11,289	2066
Resolution (Å)	72.5-2.00 (2.07-2.00)	80.0-2.00 (2.07-2.00)	72.5-2.00 (2.07-2.00)	80.0-1.90 (1.97-1.90)
Unique reflections	33,248 (3247)	8808 (844)	32,970 (3233)	10,209 (989)
Completeness (%)	100.0 (100.0)	100.0 (100.0)	100.0 (100.0)	100.0 (100.0)
Redundancy	148.5 (101.5)	126.6 (88.4)	241.9 (167.8)	158.1 (112.7)
SNR	4.01 (1.59)	4.04 (1.59)	4.19 (1.28)	3.91 (1.31)
CC	0.9755 (0.5966)	0.9637 (0.6351)	0.9817 (0.4492)	0.9672 (0.4418)
CC*	0.9937 (0.8644)	0.9907 (0.8814)	0.9953 (0.7873)	0.9916 (0.7828)
R_pslit_ (%) ^a^	17.22 (65.64)	17.06 (66.17)	18.99 (87.37)	17.70 (85.37)
Wilson B factor (Å^2^)	28.23	32.20	22.48	27.89
**Refinement**				
Resolution (Å)	70.29-2.00	56.13-2.00	71.70-2.00	56.18-1.90
R_work_/R_work_^b^	17.05/21.30	18.41/23.33	19.23/22.58	18.96/23.82
R.m.s. deviations				
Bond length (Å)	0.007	0.006	0.007	0.008
Bond angle (°)	0.860	0.838	0.836	0.970
B factors (Å)				
Protein	29.59	38.61	26.29	33.55
Ligands	38.88	47.50	40.40	41.15
Ramachandran (%)				
Preferred	95.72	95.80	96.22	95.97
Allowed	3.74	4.20	3.24	4.03
Outliers	0.53	0	0.54	0

Values for the outer shell are given in parentheses. ^a^ *R_split_* = (12)·∑hkl|Ihkleven−Ihklodd|12|Ihkleven−Ihklodd|. ^b^ *R*_work_ is defined as Σ||*F*_obs_|-|*F*_calc_||/Σ|*F*_obs_|, where *F*_obs_ and *F*_calc_ are the observed and calculated structure-factor amplitudes, respectively; and R_free_ represents the R factor for the test set (10% of the data). CC refers to Pearson’s correlation coefficient, and CC* = $\sqrt{\frac{2CC_{1/2}}{1+CC_{1/2}}}$.

**Table 2:** Chemical structure of the polysaccharide-based viscous delivery media.

Delivery Medium	Chemical Structure	Component	Reference
Agarose	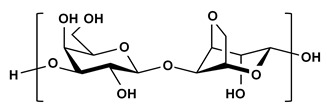	β-(1-4)-(3,6)-anhydro-L-galactose and α-(1-3)-D-galactose	[34]
Hyaluronic acid (HA)	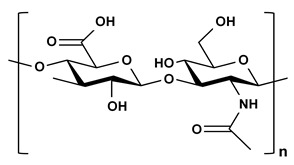	β-d-(1-3) glucuronic acid and β-d-(1-4)-N-acetylglucosamine	[32]
Hydroxyethyl cellulose (HEC)	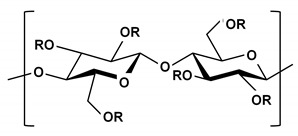	R is H or hydroxyl ethyl group (–CH_2_CH_2_–OH)	[31]
Sodium carboxymethyl cellulose (NaCMC)	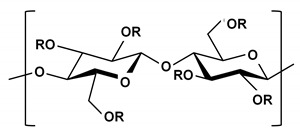	R is H or carboxymethyl groups (–CH_2_–COOH)	[35]
Wheat starch (WS)	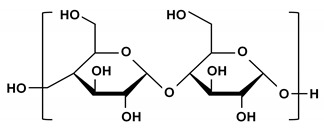	Polymer of glucose	This study
Alginate	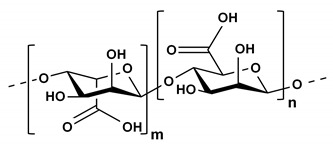	(1,4)-linked β-d-mannuronic acid and α-l-guluronic acid	This study

## Abbreviations

SX	serial crystallography
SMX	serial millisecond crystallography
SFX	serial femtosecond crystallography
WS	wheat starch
ADU	analog-to-digital unit
GI	glucose isomerase
